# Using Smartphones to Enhance Vision Screening in Rural Areas: Pilot Study

**DOI:** 10.2196/55270

**Published:** 2024-04-04

**Authors:** Zheng Wang, John Kempen, Gang Luo

**Affiliations:** 1 School of Medicine Jiaxing University Jiaxing China; 2 Schepens Eye Research Institute Massachusetts Eye and Ear Harvard Medical School Boston, MA United States; 3 Department of Ophthalmology Harvard Medical School Boston, MA United States; 4 Sight for Souls Bellevue, WA United States; 5 Eye Unit MyungSung Christian Medical Center General Hospital MyungSung Medical School Addis Ababa Ethiopia

**Keywords:** vision screening, refractive error, strabismus, smartphone, visual acuity, vision, visual, eye, eyes, screening, mHealth, mobile health, app, apps, application, applications, feasibility, optometry, ophthalmology

## Abstract

**Background:**

While it is treatable, uncorrected refractive error is the number one cause of visual impairment worldwide. This eye condition alone, or together with ocular misalignment, can also cause amblyopia, which is also treatable if detected early but still occurs in about 4% of the population. Mass vision screening is the first and most critical step to address these issues, but due to limited resources, vision screening in many rural areas remains a major challenge.

**Objective:**

We aimed to pilot-test the feasibility of using smartphone apps to enhance vision screening in areas where access to eye care is limited.

**Methods:**

A vision screening program was piggybacked on a charity summer camp program in a rural county in Sichuan, China. A total of 73 fourth and fifth graders were tested for visual acuity using a standard eye chart and were then tested for refractive error and heterophoria using 2 smartphone apps (a refraction app and a strabismus app, respectively) by nonprofessional personnel.

**Results:**

A total of 5 of 73 (6.8%, 95% CI 2.3%-15.3%) students were found to have visual acuity worse than 20/20 (logarithm of minimal angle of resolution [logMAR] 0) in at least one eye. Among the 5 students, 3 primarily had refractive error according to the refraction app. The other 2 students had manifest strabismus (one with 72–prism diopter [PD] esotropia and one with 33-PD exotropia) according to the strabismus app. Students without manifest strabismus were also measured for phoria using the strabismus app in cover/uncover mode. The median phoria was 0.0-PD (IQR 2.9-PD esophoria to 2.2-PD exophoria).

**Conclusions:**

The results from this vision screening study are consistent with findings from other population-based vision screening studies in which conventional tools were used by ophthalmic professionals. The smartphone apps are promising and have the potential to be used in mass vision screenings for identifying risk factors for amblyopia and for myopia control. The smartphone apps may have significant implications for the future of low-cost vision care, particularly in resource-constrained and geographically remote areas.

## Introduction

Visual impairment impacts individuals’ ability to participate in activities of daily living and social integration, disrupting their quality of life and health. To mitigate the impact of visual impairment, regular eye exams are important. However, regular eye exams are still a luxury for many people for various reasons that lead to limited access to eye care.

For instance, in sub-Saharan Africa, there are only 2 ophthalmologists per million people, while the global mean is 32. It is the only region in the world where the prevalence of moderate and severe vision impairment has increased from 1990 to 2020 [1,2]. Even in developed countries, disparity in eye care is a grim reality. According to US Centers for Disease Control, the percentage of American adults who reported not having an eye examination within the past year ranged from 20% to 30% [3]. The number is even higher among older persons residing in nursing homes, 53% of whom had not seen an eye care provider in the past year [4]. Inconvenience or inability to visit hospitals, cost concerns, and the limited scale of the eye care work force are the major reasons [4].

The World Health Organization and the International Agency for the Prevention of Blindness launched the VISION 2020: The Right to Sight initiative, aiming to eliminate avoidable blindness in the world by 2020 [5]. In 2021, the agency recognized that uncorrected refractive error is still a major cause of blindness and vision impairment in the world, affecting 86 million individuals [6], although it is treatable with glasses.

The rates of annual vision screening among school-aged children in developed countries are relatively high, but there are still gaps. Strabismus is one of the main risk factors for amblyopia, but it is usually not included in screening protocols due to a lack of professional skills in school nurses. When a child is flagged for amblyopia based on a stereopsis test, the vision disorder is often already present and may be difficult to treat. In 2002, the American Academy of Pediatrics strongly urged the development of efficient strabismus screening technology for preschool and school-aged children [7,8].

A number of studies and reports have suggested that community eye care programs should use health workers with entry-level qualifications and platforms to help patients in remote areas more easily access professionals; these are the 2 key approaches to the success of public vision programs [9,10]. However, a gap in these approaches is the lack of detailed vision examinations for informing medical decisions. This may cause excessively high false-positive referral rates [11]. To address this gap, we have invented several key technologies for smartphone-based vision testing, which include computer vision and psychophysical methods for measuring ocular misalignment [12,13], refractive error [14], and retinal degeneration [15]. Key features of these apps are that no specially made attachment is needed in order to use them and they run on standard smartphones. With minimal training, laypersons can use the apps to perform vision tests as long as their smartphones are compatible. The accuracy of these apps has been evaluated previously against standard clinical testing methods in hospital settings [12,14]. This paper reports the findings from a school vision screening program that used the apps we developed for the first time. We aimed to demonstrate the feasibility of an app-based vision screening program involving both strabismus and refractive error tests performed by nonprofessionals.

## Methods

### Vision Testing Apps

**Figure 1:**
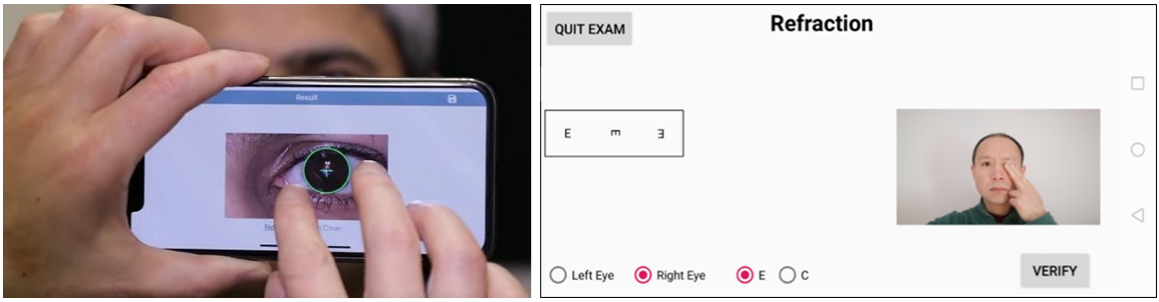
Two vision screening apps were used in this study. On the left is the photo screening app based on the Hirschberg test to measure ocular alignment. Here, the tester has zoomed in to check the visualization of the measurement. On the right is the interface of the refraction measurement app, which captures images of the patient’s face with the selfie camera and shows stimuli (the letter *E* has been chosen here) on the left of the screen according to a viewing distance estimated from the face images.

The app for strabismus assessment (Figure 1) has been validated and evaluated previously in a clinical setting by comparing it with standard clinical methods, including the modified Thorington test, the cover test with prism, and synoptophores [12]. When the app was used in this study, the automated cover test mode was used, in which one of the subject’s eyes was covered while the computer vision algorithm kept tracking the status of both eyes, and the app automatically took a snapshot of both eyes with a flash as soon as the covered eye was uncovered. This mode allowed the examiners to perform cover/uncover or alternating-cover tests. In this study, the cover/uncover test was performed. The app calculates ocular alignment using the Hirshberg method and gives results in prism diopters (PDs) [13].

The app for refractive error measurement (Figure 1) has been validated previously and evaluated in a clinical setting by comparing it with standard clinical methods, including autorefractors and noncycloplegic subjective refraction [14]. The app estimates refractive error by measuring the far point distance for discerning 20/20 Tumbling *E* letters [14] or the 20/20-equivalent clock dial chart [16]. When Tumbling *E* letters are used, the result is spherical equivalent (SE) power in diopters (D), and when the clock dial chart is used, the results give spherical power and cylindrical power based on 2 far points for perpendicular lines. In this study, the Tumbling *E* letter stimuli were used first to determine the SE, and then clock dial chart was used to estimate cylinder power around the SE range.

### Vision Screening Protocol

**Figure 2:**
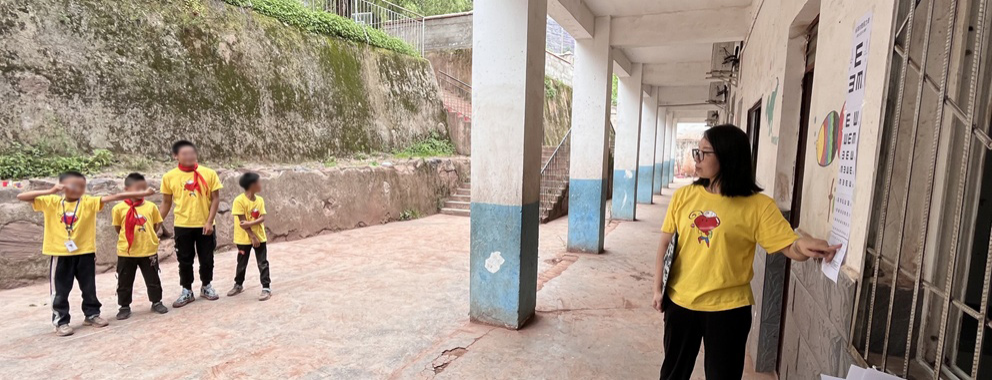
A standard Tumbling E eye chart was used in vision screening of 73 children in Sichuan, China, in 2023.

Participants first underwent visual acuity testing at a 5-m distance from a standard Tumbling *E* chart (Figure 2). There are 5 letters on each line from 20/40 downwards. Participants were given 0.02 log units credit for each extra letter read. Participants who had visual acuity worse than 20/20 in at least one eye were tested further for refractive error with the refraction app (Figure 1). Near phoria was measured with the strabismus app (Figure 1) approximately 40 cm from the phone while the participants were instructed to fixate on the phone flash. The vision measurement with apps was performed by personnel without optometry expertise. All vision tests were performed under a noncycloplegic condition.

### Participants

A total of 73 fourth and fifth graders in Meigu County, Sichuan, China, who attended a charity summer camp in 2023 were screened for vision as a part of the charity program. All children lived locally and were members of the Yi people, an ethnic minority group in China. The per capita gross domestic product in Meigu County is about US $2270, 18% of the Chinese national average. Being in a low-income mountainous area, most of the children had never undergone vision testing due to very limited access to local eye care services. They needed to be taught how to report the orientation of the letter *E* on the eye chart.

### Ethical Considerations

The vision screening was one component of the charity summer camp program. Consent for the vision test was included in the summer camp consent form. Participants received free tutoring classes, meals, and school supplies during the 2-week summer camp. Other than that, they were not compensated specifically for participating in the vision screening. This paper reports the results of a secondary data analysis. The vision measurement data, with individual identifiable information removed, were shared by the charity program. This research received approval with exemption of consent from the Institutional Review Board of Massachusetts Eye and Ear. The study was conducted in accordance with the tenets of the Declaration of Helsinki.

## Results

We identified 5 students (7%, 95% CI 2.3%-15.3%) as having visual acuity worse than 20/20 in at least one eye (Table 1). Measuring refraction with the app showed that 2 students had astigmatism (patient P1, cylinder –1 D in the left eye, patient P4, cylinder –0.5 D in the right eye). One student was found to be hyperopic in both eyes (patient P3, sphere +3.2 D in both eyes), and also was strabismic (72-PD esotropia). This was probably a case of accommodative esotropia [17]. His refractive error was not measured directly at the site, but estimated based on the commonly used near point distance of human eyes (25 cm). According to the app, his near point was at 1.2 m, which corresponds to –0.8 D, where he could not see 20/80 letters at close range and maintained 20/80 vision beyond the near point. Therefore, the estimate of his refractive error was 4 – 0.8 = 3.2 D. According to Mäntyjärvi’s [18] findings in a hyperopic population, the amplitude of accommodation is at least 4 D, based on which the estimate would also be 3.2 D. Another student was also found to be strabismic (patient P4, 35-PD exotropia). The 2 students with manifest strabismus were suspected to have amblyopia. The student with the worst visual acuity among the participants (20/100 in the right eye and 20/167 in the left eye) had moderate myopia (patient P5, sphere –3 D in both eyes), and her near-phoria was 7-PD esotropia. This student was given a pair of off-the-shelf glasses (–3 D for both eyes), and her vision in both eyes improved to 20/33.

**Table 1:** Among the 73 students screened in a rural area in China, participants who had visual acuity lower than 20/20 in at least 1 eye (5 of 73 children) are listed here. Refraction and phoria were measured using the 2 apps shown in Figure 1. The text describes the estimation method.

Patient	Visual acuity 20/xx (logMAR^a^)			Refraction (diopters)			Phoria/tropia (prism diopters)
	Right eye	Left eye	Right eye		Left eye		
P1	20 (0)	25 (0.1)	Sphere –0.5		Sphere –1.2; cylinder –1.0	4 esophoria	
P2	17 (–0.07)	26 (0.12)	Sphere –0.8		Sphere –0.9	2 exophoria	
P3^b^	80 (0.6)	80 (0.6)	Sphere +3.2		Sphere +3.2	72 esotropia^c^	
P4	33 (0.2)	25 (0.1)	Sphere 0.5; cylinder –0.5		Sphere –0.5	33 exotropia^c^	
P5	100 (0.7)	167 (0.9)	Sphere –3.0		Sphere –3.0	7 esophoria	

^a^logMAR: logarithm of minimal angle of resolution.

^b^Refraction for patient 3 was calculated based on the near point acquired by the refraction app.

^c^Manifest strabismus, measured without cover.

**Figure 3:**
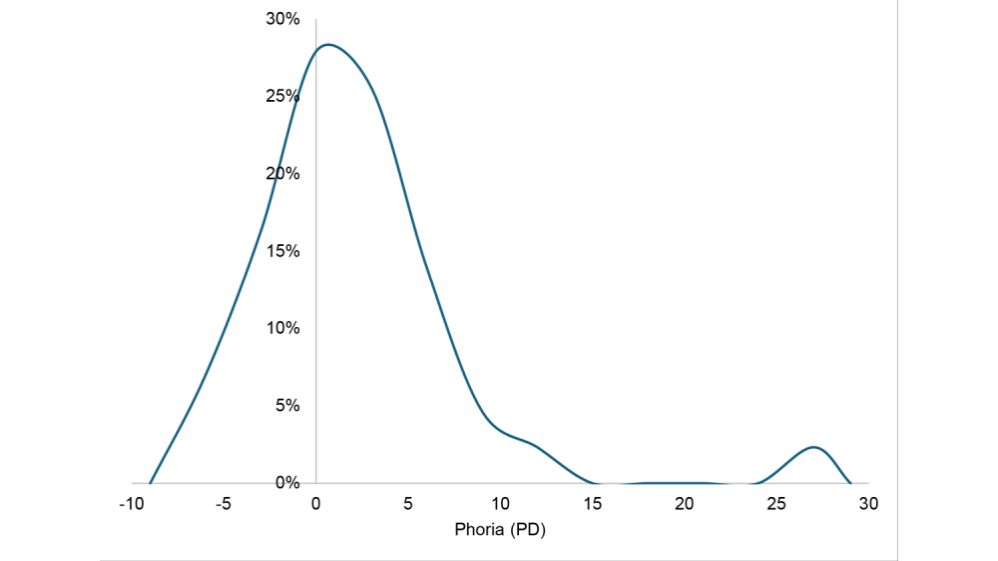
Distribution of phoria measured with the strabismus app during vision screening. Negative values represent exophoria. The curve is smoothed by a 3-PD wide sliding window. PD: prism diopter.

Figure 3 shows the distribution of the near-phoria of the students, excluding the 2 students with manifest strabismus, who are listed in Table 1. Phoria was under 10 PD in the majority. The median phoria was 0.0 PD (IQR 2.9-PD esophoria to 2.2-PD exophoria). There was 1 outlier, a student with 26-PD esophoria and normal visual acuity. Excluding this outlier, the mean phoria of the group was 0.2 (SD 4.1)-PD esophoria.

## Discussion

### Summary

By using 2 mobile apps designed for vision testing, ocular alignment and refraction measurements were successfully and easily incorporated in school vision screening. The testing results provided much richer information than conventional vision screening, which normally does not include the 2 tests. Based on this particular screening, the vision issues among the rural-area, school-aged children were primarily uncorrected refractive error and amblyopia.

### Interpretations

While myopia prevalence rates are very high in East Asia, including China, where in some economically developed cities, it is even above 80% among school-aged students [19], the majority of the fourth and fifth grade students in this screening had emmetropic vision (uncorrected visual acuity better than 20/20). Ample outdoor activity time and a light burden of study requiring the use of near vision, as we witnessed and surveyed during the summer camp, are likely the main reasons for the large differences between these students and their urban counterparts. Nevertheless, 4% (3/73) of the children mainly had refractive error (patients P1, P2 and P5 in Table 1). This rate is very similar to the prevalence (4.1%) found in the rural child population in India [20]. In addition, there were 2 students (2.7%) with manifest strabismus (patients P3 and P4 in Table 1). This rate is within the range of strabismus prevalence (2.06%-3.9%) among children in Taiwan in the years 2014 to 2019 [21]. As for the phoria of the nonstrabismic students in this study (mean phoria 0.2-PD esophoria, SD 4.1 PD; IQR 2.9-PD esophoria to 2.2-PD exophoria), it is similar to the normative range found among 879 elementary school students by Lyon et al [22] using the modified Thorington test (mean 1-PD exophoria, SD 4 PD; IQR 2-PD esophoria to 2-PD exophoria).

The consistency with epidemiology findings from other large population-based vision screening studies suggests that the screening results in this study are probably reliable. A difference from previous screening studies, though, is that this screening study used smartphone-based strabismus and refractive error tests, while most vision screening studies were conducted by professionals using conventional optometry methods. For instance, in the screening study by Dandona et al [20], strabismus was assessed using a penlight and refractive error was measured using a streak retinoscope and handheld autorefractor; both measurements were made by ophthalmic professionals. Our study provides a proof of concept that vision screening tests, including for strabismus and refractive error, can be performed using smartphone apps by persons without optometry expertise. A further study to develop the use of this simple and portable approach to mass vision screening is warranted.

### Limitations

One limitation of this study was that the vision test results were not verified by clinical gold standard testing due to the nature of the education charity program. However, it should be noted that the main focus of this study was not to evaluate measurement accuracy, as previous evaluation studies have already addressed that aspect. The difference from our previous evaluation is that this study involved a sample that included people with and without eye conditions, while previous studies deliberately selected patients with a wide range of conditions.

### Implications

Thanks to their affordability, ubiquity, and validity, smartphone-based vision screening tools have the potential to play a pivotal role in mass vision screening efforts, particularly in areas where access to eye care is limited. These innovative tools may impact eye health care by providing a cost-effective and convenient solution for screening, identifying, and addressing vision issues, especially in underserved communities.

One example of such technology is the Peek Acuity app (Peek Vision), which has made significant strides in the field of mobile vision screening. This app has been deployed to screen tens of thousands of individuals in some studies in Africa [9,23,24]. Its success in large-scale vision screening efforts underscores the scalability and effectiveness of smartphone-based apps. By offering an efficient way to measure visual acuity, the Peek Acuity app has empowered health care providers to reach more people, making it a useful tool for addressing visual impairment.

Another photoscreening app, GoCheck Kids (Gobiquity Inc), is primarily designed to assess for amblyopia risk factors [11,25]. By analyzing eccentric photorefraction red-reflex images, the app does not require patients to respond, and it therefore can be used to examine preverbal children. The app has been tested in clinical trials by comparing it with its counterparts among dedicated screening devices, such as iScreening [26]. It is reported by the company that developed GoCheck Kids that 4 million children have been screened [27].

Considering the successful deployment of these smartphone apps, we expect that a mobile technology–based vision screening approach, operated by personnel with basic training, will be scalable and will complement service programs provided by ophthalmic clinicians and dedicated screening devices (eg, iScreen, Spot Vision Screener). The mobile app approach is particularly suitable for low -resource countries and could also be used for screening in classrooms. Compared to Peek Acuity and GoCheck Kids, the screening apps assessed in this study can measure refractive error and strabismus quantitatively rather than flagging for risk factors (GoCheck Kids) or not measuring these most common conditions at all (Peek Acuity).

Our future investigations will aim to assess the positive and negative predictive values of these apps in large-scale vision screening programs, building upon the feasibility demonstrated in this preliminary study.

## Abbreviations

D: diopter
PD: prism diopter
SE: spherical equivalent

## Data Availability

Data used in this paper may be made available for research purposes if requested through the corresponding author.
